# Morphological variation of freshwater crabs *Zilchiopsis collastinensis* and *Trichodactylus borellianus* (Decapoda, Trichodactylidae) among localities from the middle Paraná River basin during different hydrological periods

**DOI:** 10.3897/zookeys.457.6726

**Published:** 2014-11-25

**Authors:** María Victoria Torres, Pablo Agustín Collins, Federico Giri

**Affiliations:** 1 Instituto Nacional de Limnología (CONICET-UNL). Ciudad Universitaria Paraje El pozo s/n, Santa Fe, Argentina; 2 Facultad de Bioquímica y Ciencias Biológicas (UNL). Ciudad Universitaria Paraje El pozo s/n, Santa Fe, Argentina; 3 Facultad de Humanidades y Ciencias, (UNL), Ciudad Universitaria Paraje El pozo s/n, Santa Fe, Argentina

**Keywords:** Geometric morphometrics, Brachyura, floodplain, connectivity

## Abstract

Measures of hydrologic connectivity have been used extensively to describe spatial connections in riverine landscapes. Hydrologic fluctuations constitute an important macrofactor that regulates other environmental variables and can explain the distribution and abundance of organisms. We analysed morphological variations among individuals of two freshwater crab species, *Zilchiopsis collastinensis* and *Trichodactylus borellianus*, from localities of the middle Paraná River basin during two phases of the local hydrological regime. Specimens were sampled at sites (localities) of Paraná River, Saladillo Stream, Salado River and Coronda River when water levels were falling and rising. The conductivity, pH, temperature and geographical coordinates were recorded at each site. The dorsal cephalothorax of each crab was represented using 16 landmarks for *Zilchiopsis collastinensis* and 14 landmarks for *Trichodactylus borellianus*. The Canonical Variate Analyses showed differences in shape (for both species) among the crabs collected from the Paraná and Salado Rivers during the two hydrologic phases. We did not find a general distribution pattern for shape among the crab localities. During falling water, the shapes of *Zilchiopsis collastinensis* were not related to latitude-longitude gradient (i.e., showing greater overlap in shape), while during rising water the shapes were ordered along a distributional gradient according to geographical location. Contrary, shapes of *Trichodactylus borellianus* were related to latitude-longitude during falling water and were not related to distributional gradient during rising water. The cephalothorax shape showed, in general, no statistically significant covariations with environmental variables for either species. These results show that each freshwater crab species, from different localities of the middle Paraná River, remain connected; however, these connections change throughout the hydrologic regime of the floodplain system. This study was useful for delineating how the relation among shapes of crabs of localities varies during two phases of the hydrological regime and for estimating the connections and geographical patterns in the floodplain system.

## Introduction

Measures of hydrologic connectivity have been used extensively to describe spatial connections in riverine landscapes (Ward 1989, Amoros and Bornette 2002). The dynamic and hierarchical nature of lotic ecosystems can be conceptualised as a four-dimensional fluvial hydrosystem, displaying distinct longitudinal, lateral, vertical and temporal characteristics (Ward 1989).

Floodplain systems vary broadly in their environmental characteristics and hydrological regimes (Ward et al. 2002). The alluvial valley, principally located in the middle and lower section of Paraná River, is a complex ensemble of lotic and lentic environments (Drago 2007). The main factor modelling the dynamics of the Paraná River system and its floodplain is the hydro-sedimentological cycle (Junk et al. 1989, Neiff 1990). Rising and falling water make two complementary phases of the cycle, which have much influence on the stability of river ecosystems (Neiff 1990). The period of flood is typically during spring and summer, while the period of low water is in autumn and winter (Drago 2007). In this sense, this hydrological fluctuation constitute an important macrofactor that regulates environmental variables and can explain the richness, distribution and abundance of organisms that live in these systems (Junk et al. 1989, Neiff et al. 2001, Mayora et al. 2013).

The movements of some species are related to the spatial and temporal dynamics of the floodplain system that they inhabit. Dispersal is defined as the movement of an organism over a specified distance or from one predefined patch to another (Bennetts et al. 2003). Depending on the hydrologic regime of the floodplain system, dispersal tends to homogenise populations between water bodies during high water periods and to decrease the differences between richness and abundance of organisms at nearby sites (Gomes et al. 2012). Freshwater invertebrates disperse through active or passive movements, which can influence colonisation rates, gene flow, and evolutionary divergence (Bilton et al. 2001).

The movements of freshwater decapods are influenced by biotic and abiotic factors in dynamic floodplain systems, and these factors vary over different spatial and temporal scales (Williner et al. 2010). The regular movements of floodplain decapods are typically active or passive displacements within lakes, ponds or rivers (Williner et al. 2009). Little is known about the dispersal of the large freshwater crab *Zilchiopsis collastinensis* (Pretzmann, 1968). This burrowing crab spends most of its life on river banks in canyons where the locations of caves varies with the river’s water level (Williner et al. 2009). More is known about the smaller freshwater crab *Trichodactylus borellianus* Nobili, 1896, which inhabits the roots of water hyacinths and moves passively with macrophyte displacements (Collins et al. 2006).

The dispersal and connectivity of freshwater invertebrate populations are difficult to study directly. One way to perform such studies is by assessing the differences or similarities in the shape of the organisms between populations. Morphometric studies are useful for delineating the shapes of various populations and species over geographical ranges and such studies can provide evidence of regional differences in crustaceans (Rufino et al. 2006, Konan et al. 2010, Silva et al. 2010, Srijaya et al. 2010, Bissaro et al. 2013). Geometric morphometrics (GM) can be used to quantify the variation in these forms (Monteiro 1999), generating a set of shape variables that can be used to test statistical hypotheses and providing a means of visually describing patterns of shape differences in the data (Rohlf and Marcus 1993, Adams 1999). Previous studies have used GM to compare populations of freshwater decapods (Giri and Collins 2004, Giri and Loy 2008, Barría et al. 2011, Idaszkin et al. 2013). The aim of this paper was to study and infer about the population connectivity of *Zilchiopsis collastinensis* and *Trichodactylus borellianus* in the context of a floodplain system, through the analysis the morphological variations observed among crabs from localities of the middle Paraná River during two phases of their habitat’s hydrological regime.

## Methods

**Figure 1.**
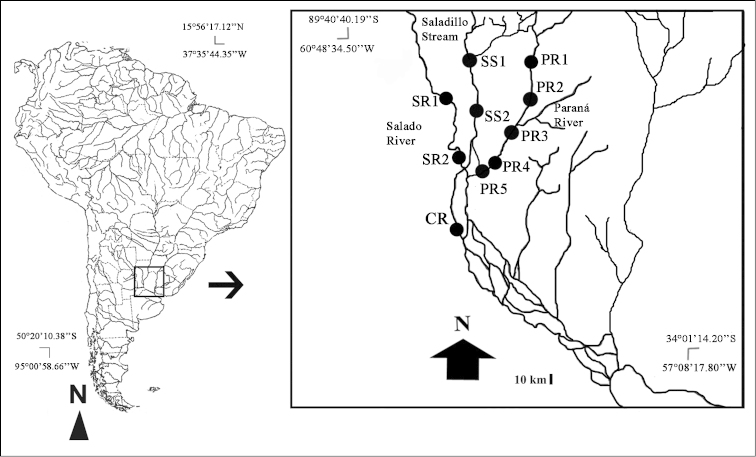
Sampling sites (localities): Paraná River (PR1, PR2, PR3, PR4, PR5); Saladillo Stream (SS1, SS2); Salado River (SR1, SR2); Coronda River (CR).

*Zilchiopsis collastinensis* and *Trichodactylus borellianus* were collected from macrophytes using hand nets and from caves by hand (*Zilchiopsis collastinensis* only). Samples were collected from sites (localities) along the Paraná River (PR1, PR2, PR3, PR4 and PR5), the Saladillo Stream (SS1 and SS2), the Salado River (SR1 and SR2) and the Coronda River (CR) (Fig. 1). After collection, the specimens were chilled and then preserved with 96% ethanol for further Geometric Morphometrics (GM) analysis. The individuals collected were deposited in the Laboratorio de macrocrustaceos del Instituto Nacional de Limnología (INALI-CONICET-UNL).

**Figure 2.**
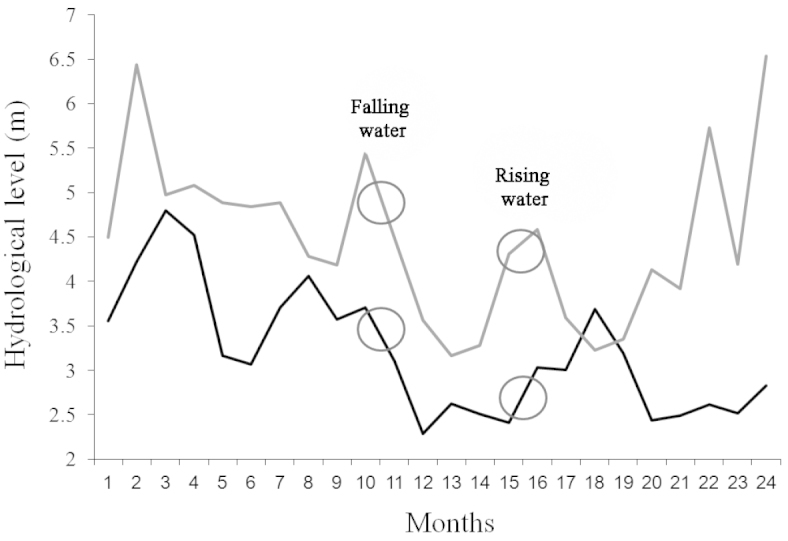
Different phases of the hydrological regime according to hydrological level. Paraná River (black line) and Salado River (gray line). The circles indicate the water level in which the crabs were collected from each river (falling and rising water).

Each site was sampled during two different phases of the hydrological regime (i.e., when water levels were falling and rising in the Paraná and Salado River) (Fig. 2). Data on hydrometric levels were obtained from local ports and from the Facultad de Ingeniería y Ciencias Hídricas (Universidad Nacional del Litoral). In addition, conductivity, pH and temperature were measured at each site with a digital sensor (HANNA 198130) (Table 1). The geographical locations according to geographic coordinates of the sampling sites were obtained using GPS tracking (Garmin Dakota 20).

**Table 1.** Environmental variables measured in each sample site during two hydrological phases of the middle Paraná River basin. Paraná River (PR1, PR2, PR3, PR4 and PR5); Saladillo Stream (SS1, SS2); Salado River (SR1, SR2); Coronda River (CR).

Sampling sites	Falling water			Rising water		
	Conductivity (µS cm^-1^)	Temperature (°C)	pH	Conductivity (µS cm^-1^)	Temperature (°C)	pH
PR1	130	27.4	8.29	130	23.7	7.78
PR2	90	21.2	8.31	120	17.5	8.25
PR3	60	26.8	7.82	130	23.3	8.25
PR4	90	21.6	8.02	-	-	-
PR5	80	22	8.1	-	-	-
SS1	760	26.1	8.02	-	-	-
SS2	-	-	-	1340	20.3	7.82
SR1	-	-	-	2680	21.3	7.9
SR2	1470	24.1	7.87	4680	23.2	7.99
CR	390	25.7	7.94	370	23.7	8.21

**Figure 3.**
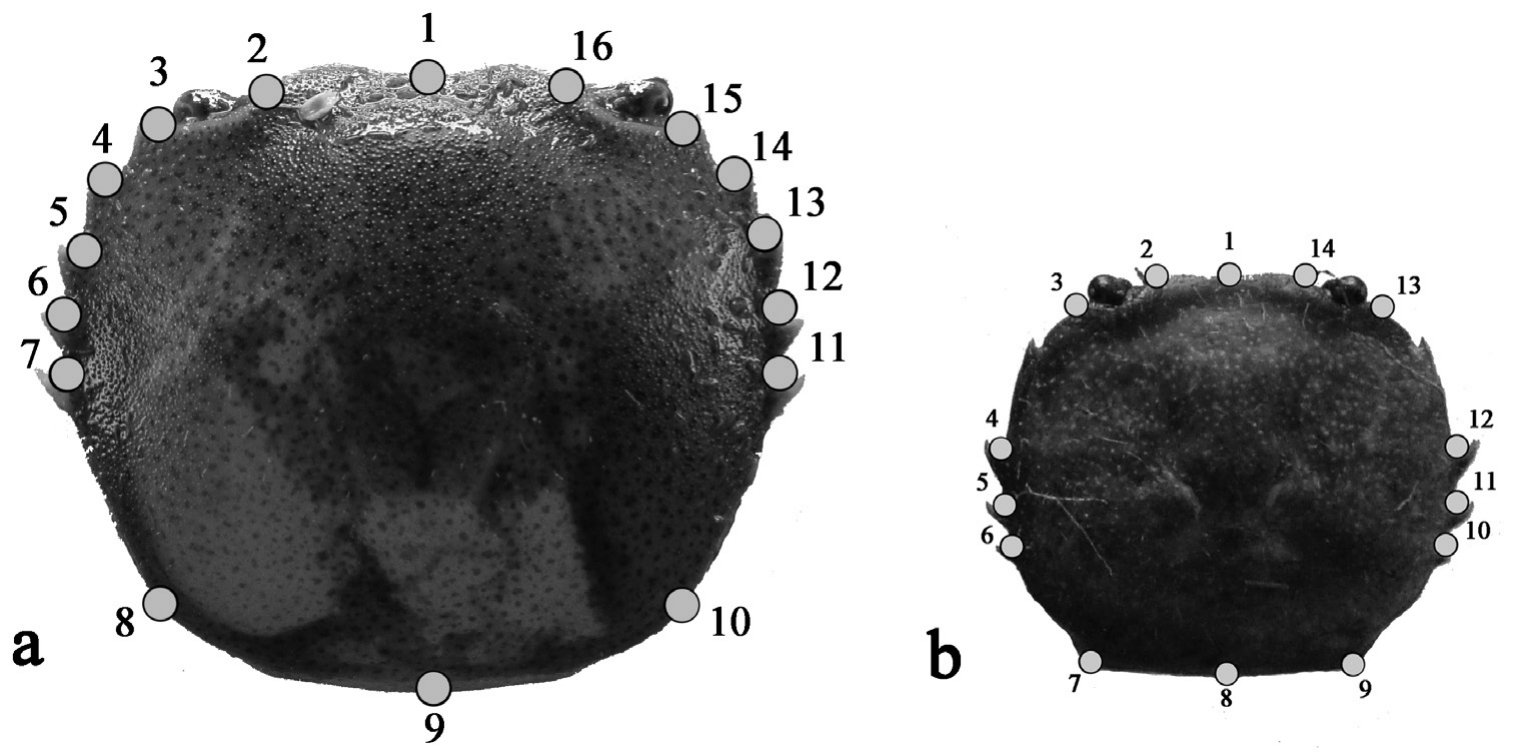
Cephalothorax with configuration of 16 landmarks on *Zilchiopsis collastinensis* (**a**) and 14 landmarks on *Trichodactylus borellianus* (**b**).

To apply the GM analysis, digital images of each crab’s cephalothorax were taken using a Sony Cyber-shot digital camera with a 12.1 mp resolution. The cephalothorax structure of *Zilchiopsis collastinensis* was represented using 16 digitised landmarks (Type I: LMs #2 to 7; LMs #11 to 16 and Type II: LMs #1, 8, 9 and 10) (Bookstein 1991) (Fig. 3a). For *Trichodactylus borellianus*, 14 landmarks were digitised (Type I: LMs #2 to 7; LMs #9 to 14 and Type II: LMs #1 and 8) (Fig. 3b). The computer program tpsDig 1.40 was used to digitise these landmarks (Rohlf 2004).

Following the GM analysis, the shape symmetric components associated with position, rotation, translation and size were removed using the Procrustes fit in the program MorphoJ (Klingenberg 2011). Variation in the shape symmetric components was explored via a principal component analysis (PCA) applied to the Procrustes coordinates. Intrapopulation allometry was tested using multivariate regression, with centroid size as the independent variable. The size correction was made by taking into account the residuals of the common pooled within-group regression in the program MorphoJ. Sexual dimorphism was also tested. However, the results ultimately showed that male and female crabs displayed similar variation in shape among localities for both species, and therefore samples were not segregated by sex in subsequent analyses.

Permutations were used to establish the significance of each statistical test, employing 10,000 permutations for the multivariate regression (Klingenberg 2011).

Variations in the shape symmetric component among sites for each moment of the hydrologic regime were analysed using Procrustes pairwise permutation tests and Canonical Variate Analyses (CVA) with the program MorphoJ (10,000 permutations), with residuals of the pooled within-group regression as the focal dataset.

The covariations among shapes, environmental variables and geographical location (altitude and longitude) were analysed with the software tpsPLS (Rohlf 2006) using a permutation test with 99 randomisations. This program uses a two-block partial least-squares analysis, calculating the covariation and correlation between shape and a set of variables (Rohlf 2006). We used this type of analysis to examine whether shape among crabs of different localities varied as a result of certain environmental variables or geographical location in each river during the two phases of the hydrological regime.

## Results

The number of specimens collected and analyzed differed for each site depending on the phase of the hydrological regime (Table 2). The geographical locations of the sampling sites were relatively close together (Table 2).

**Table 2.** Number of specimens collected (*Zilchiopsis collastinensis* and *Trichodactylus borellianus*) and analyzed during two different phases of the hydrological regime of the middle Paraná River basin. Paraná River (PR1, PR2, PR3, PR4 and PR5); Saladillo Stream (SS1, SS2); Salado River (SR1, SR2); Coronda River (CR).

Sampling Sites	Geographical location		Falling water		Rising water	
	latitude	longitude	*Zilchiopsis collastinensis*	*Trichodactylus borellianus*	*Zilchiopsis collastinensis*	*Trichodactylus borellianus*
PR1	30°35'01.07"S	59°56'58.31"W	8	-	5	7
PR2	31°10'06.50"S	60°08'16.87"W	-	21	11	-
PR3	31°35'13.51"S	60°33'06.06"W	-	13	8	-
PR4	31°38'33.31"S	60°40'35.83"W	-	20	-	11
PR5	31°39'02.96"S	60°40'29.45"W	20	-	-	-
SS1	30°27'03.45"S	60°05'37.14"W	-	13	-	-
SS2	31°16'44.69"S	60°33'25.10"W	-	-	-	27
SR1	30°59'58.47"S	60°49'48.11"W	-	-	7	-
SR2	31°37'30.11"S	60°45'42.32"W	32	23	-	17
CR	31°43'32.93"S	60°45'22.47"W	6	25	14	12
Total			**66**	**115**	**45**	**74**

Variation in shape was ordered along PC1 by site at the two hydrological periods for *Zilchiopsis collastinensis* (PC1: 72.42% and PC2: 10.00% when water levels were falling; PC1: 74.53% and PC2: 6.98% when water levels were rising) and along PC2 by site for *Trichodactylus borellianus* (PC1: 44.62% and PC2: 15.72% when water levels were falling; PC1: 41.55% and PC2: 16.59% when water levels were rising).

All crabs from all localities of both species exhibited significant (*p* < 0.05) allometric relationships between cephalothorax shape and centroid size during two phases of the hydrologic regime.

**Figure 4.**
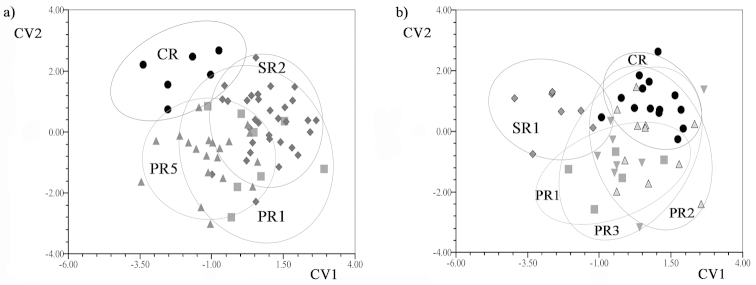
Graphics of Canonical Variate Analyses (CVA) of cephalothorax shapes of *Zilchiopsis collastinensis* between localities. Ellipses represent the confidence interval at 90%. Paraná River (PR1, PR2 PR3, PR5); Salado River (SR1, SR2); Coronda River (CR). **a** falling water **b** rising water.

**Figure 5.**
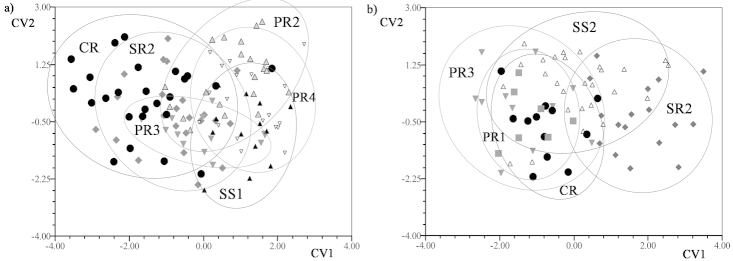
Graphics of Canonical Variate Analyses (CVA) of cephalothorax shapes of *Trichodactylus borellianus* between localities. Ellipses represent the confidence interval at 90%. Paraná River (PR1, PR2 PR3, PR4); Saladillo Stream (SS1, SS2); Salado River (SR2); Coronda River (CR). **a** falling water **b** rising water.

Differences in shape variation were observed among the crabs of the Paraná and Salado Rivers during the two phases of the hydrologic regime (Figs 4 and 5) (Procrustes pairwise permutation tests with CVA). For *Zilchiopsis collastinensis*, individuals of localities in the Paraná River were similar in shape when water levels were falling. Additionally, individuals collected from PR1 had shapes similar to those collected in SR2 (Salado River site) (Fig. 4a) (Table 3) and in CR (Fig. 4a) (Table 3). When water levels were rising, individuals collected from localities in the Paraná River were similar in shape (Fig. 4b) (Table 3). No differences in shape were observed among individuals in localities in both rivers compared to those in CR (Fig. 4b) (Table 3). Individuals collected from both rivers were more similar in shape when water levels were falling than when water levels were rising (Fig. 4a and b). When water levels were rising, crabs collected from localities in the Paraná River had overlapping shapes but these shapes were different from those of crabs collected from the Salado River site (Fig. 4b).

**Table 3.** Procrustes pairwise permutation tests with Canonical Variate Analyses (CVA) of cephalothorax shapes of *Zilchiopsis collastinensis* and *Trichodactylus borellianus* between localities, during two different phases of the hydrological regime of the middle Paraná River basin. The upper right triangle gives the Procrustes distances and the lower left triangle gives the *p*-values from permutation tests for Procrustes distances among shapes of crabs from localities. Paraná River (PR1, PR2, PR3, PR4 and PR5); Saladillo Stream (SS1, SS2); Salado River (SR1, SR2); Coronda River (CR).

***Zilchiopsis collastinensis***						
**Falling water**	PR1	PR5	SR2	CR		
PR1	-	0.0104	0.0081	0.0189		
PR5	0.3619	-	0.0107	0.0191		
SR2	0.2779	0.0065**	-	0.0162		
CR	0.0699	0.0402*	0.0069**	-		
**Rising water**	PR1	PR2	PR3	SR1	CR	
PR1	-	0.0125	0.0115	0.0129	0.0134	
PR2	0.0904	-	0.0143	0.0166	0.0081	
PR3	0.2474	0.0172*	-	0.0131	0.0128	
SR1	0.2887	0.0119*	0.1801	-	0.0128	
CR	0.1262	0.3750	0.0642	0.1193	-	
*Trichodactylus borellianus*						
**Falling water**	PR2	PR3	PR4	SS1	SR2	CR
PR2	-	0.0158	0.0109	0.0101	0.0127	0.0163
PR3	0.017*	-	0.0185	0.0167	0.0185	0.0173
PR4	0.0771	0.0057*	-	0.007	0.0147	0.0204
SS1	0.1714	0.0178*	0.64	-	0.0147	0.0206
SR2	0.0136*	0.0021**	0.0038**	0.0054*	-	0.0090
CR	0.002**	0.0097*	0.0001***	0.0002***	0.1882	-
**Rising water**	PR1	PR3	SS2	SR2	CR	
PR1	-	0.0097	0.0133	0.0182	0.0088	
PR3	0.4162	-	0.0146	0.0196	0.0109	
SS2	0.0656	0.0112*	-	0.0098	0.0095	
SR2	0.0006***	<.0001***	0.0645	-	0.0163	
CR	0.4218	0.1778	0.1623	0.0009***	-	

Statistically significant differences, * *p* < 0.05, ** *p* < 0.005, *** *p* < 0.001.

For *Trichodactylus borellianus*, despite some differences in the results of the CVA for samples collected when water levels were falling, individuals from localities in the Paraná River were similar in shape (Fig. 5a) (Table 3). These were different in shape compared to those in the CR site (Fig. 5a) (Table 3). Shape variation differed when water levels were rising, being the crabs from localities in the Paraná River similar in shape to crabs from CR (Fig. 5b) (Table 3). For both phases of the hydrologic regime, crabs from localities in the Paraná River had overlapping shapes, but those shapes were different from those for crabs in SR2 (Fig. 5a and b).

**Figure 6.**
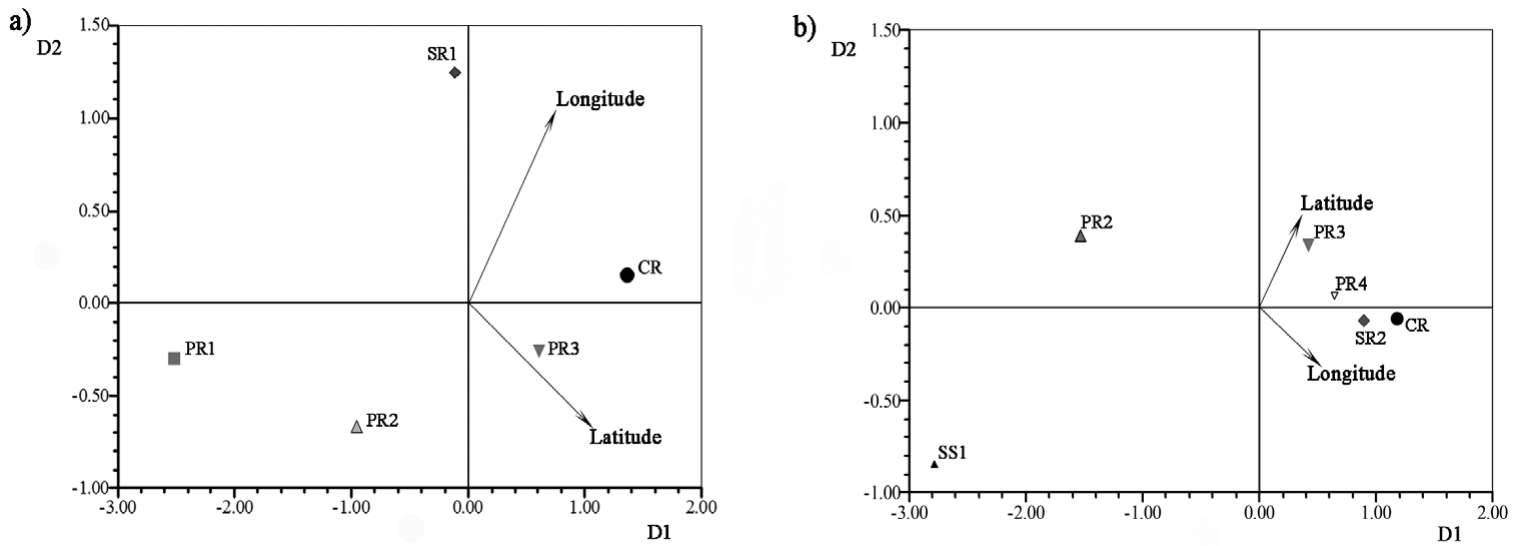
Results of the tpsPLS applying the two-block partial least-squares analysis. **a** *Zilchiopsis collastinensis* when water levels were rising **b** *Trichodactylus borellianus* when water levels were falling. Paraná River (PR1, PR2 PR3, PR4); Saladillo Stream (SS1); Salado River (SR1; SR2); Coronda River (CR).

*Zilchiopsis collastinensis* and *Trichodactylus borellianus* presented particular shape variations that were related to geographical location during the two phases of the hydrologic regime. For instance, the covariation between shape and distribution of *Zilchiopsis collastinensis* when water levels were falling was statistically not significant. In this case, individuals from localities in the Paraná River and the Salado River were more similar in shape, as revealed by the CVA (Fig. 4a) (Table 4). However, significant high covariation between cephalothorax shape and geographical location was observed when water levels were rising (Table 4). Crabs from localities along the Paraná River were ordered by shape according latitude-longitude gradient and this pattern was separate from that of individuals from the site of the Salado River (Fig. 6a). In contrast, *Trichodactylus borellianus* displayed significant covariation between shape and latitude-longitude when water levels were falling (Table 4), and individual shapes were ordered along a distributional gradient for localities in both rivers (Fig. 6b). Covariation between shape and geographical location was not statistically significant for samples collected when water levels were rising (Table 4). For both species, the crabs of closest sites (based on latitude and longitude) were more similar in shape when covariation was significant (Table 4) (Fig. 6a and b). However, this pattern of covariation was not observed for the two hydrological periods for the two species.

**Table 4.** *Zilchiopsis collastinensis* and *Trichodactylus borellianus*: covariations among crab cephalothorax shapes of localities and geographical location during two phases of the hydrological regime of the middle Paraná River basin.

Geographical location	Cephalothorax shape			
	*Zilchiopsis collastinensis*		*Trichodactylus borellianus*	
**Falling water**	*%Cov.*	*p-value*	*%Cov.*	*p-value*
Dimension 1	0.9954	0.83	0.9898	0.03*
Dimension 2	0.0045	0.18	0.0101	0.98
**Rising water**	*%Cov.*	*p-value*	*%Cov.*	*p-value*
Dimension 1	0.9835	0.02*	0.9547	0.79
Dimension 2	0.0164	0.99	0.0452	0.22

Statistically significant differences, *p < 0.05.

**Figure 7.**
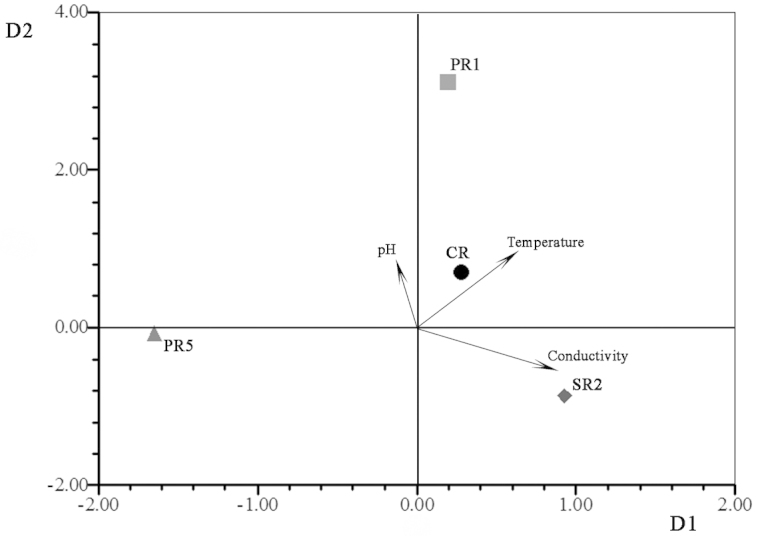
Results of the tpsPLS applying the two-block partial least-squares analysis on *Zilchiopsis collastinensis* when water levels were falling. Paraná River (PR1, PR5); Salado River (SR2); Coronda River (CR).

Cephalothorax shape was not significantly related to environmental variables for either species (Table 5), with the exception of when water levels were falling for localities of *Zilchiopsis collastinensis*. At this time, conductivity was most related to shape (Fig. 7).

**Table 5.** *Zilchiopsis collastinensis* and *Trichodactylus borellianus*: covariations among crab cephalothorax shapes of localities and environmental variables during two phases of the hydrological regime of the middle Paraná River basin.

Environmental variables	Cephalothorax shape			
	*Zilchiopsis collastinensis*		*Trichodactylus borellianus*	
**Falling water**	*%Cov.*	*p-value*	*%Cov.*	*p-value*
Dimension 1	0.9769	0.01*	0.7321	0.47
Dimension 2	0.9984	0.03*	0.9708	0.20
**Rising water**	*%Cov.*	*p-value*	*%Cov.*	*p-value*
Dimension 1	0.9020	0.16	0.7050	0.53
Dimension 2	0.9760	0.23	0.9702	0.14

Statistically significant differences, *p < 0.05.

## Discussion

The relation of cephalothorax shape among localities of two freshwater crabs (*Zilchiopsis collastinensis* and *Trichodactylus borellianus*) collected from connected rivers was different during two phases of the rivers’ hydrological regimes. In this ecological system, whether water levels were falling or rising impacted the population connectivity for the two species. This suggests that individuals were interchanged among localities by dynamic processes of the rivers. Generally, rivers in floodplain systems exhibit considerable heterogeneity that varies over multiple temporal and spatial scales (Neiff et al. 2001). Variability over short time scales, such as seasonal flooding, affects the viability of in-stream populations through changes in recruitment, survival and dispersal (Poff et al. 1997). In addition, hydrological connectivity plays an important role in the movement of populations by connecting various landscape patches (Ward 1989, Amoros and Bornette 2002, Pringle 2003).

According to the phases when crabs were more similar in shape (falling water for *Zilchiopsis collastinensis* and rising water for *Trichodactylus borellianus*), this can be related to the movements through to a dynamic floodplain system. Generally, water flow patterns become more important in systems with floodplains because currents have an effect on faunal distribution and on the movement of aquatic invertebrates (Olden et al. 2004, Grönroos et al. 2013). As a result, the movements of freshwater decapods are induced by both biotic and abiotic factors in a dynamic floodplain system and these movements can occur over different spatial and temporal scales (Williner et al. 2010). The freshwater crab *Trichodactylus borellianus* moves passively as a function of macrophyte migrations (Collins et al. 2006). *Zilchiopsis collastinensis* was also found to be associated with macrophytes in this study. Water flow within the hydrological regime is one of the primary factors regulating the growth and distribution of aquatic plants in streams and rivers and affects the passive movements of crustaceans (Chambers et al. 1991). This was also reported by Schiesari et al. (2003), who documented the potential role of macrophyte rafts in the dispersal of organisms across banks and possibly over very large distances in Amazonian rivers.

We did not find a general distribution pattern for crab localities at the two phases of the hydrologic regime. Shapes of *Zilchiopsis collastinesis* were not related to location during falling water, while shapes of *Trichodactylus borellianus* were not related to location during rising water. This would imply that the crabs’ morphological characteristics were not related to latitude-longitude, with high overlap in shape of crabs among the various localities irrespective the origin of the river. Thus, crabs of even distant localities had similar characteristics in shape. In this sense, the flow of water currents becomes particularly important in floodplain systems because the flow regime organises the river ecosystem and strongly affects population dynamics (Poff et al. 1997, Neiff et al. 2001). Four different density stages were observed for palaemonids and trichodactilids in the Middle Paraná River, coinciding with events in the hydrological cycle (Collins et al. 2006, Williner et al. 2010). Additionally, population increases for the prawn *Macrobrachium amazonicum* Heller, 1862 in the Amazon River was associated with prawn migrations during floods (Walker and Ferreira 1985). Furthermore, the hydrologic regime of a floodplain system tends to homogenise populations between water bodies during high water periods, attenuating the differences between populations (Gomes et al. 2012). On the other hand, during rising water, the shapes of *Zilchiopsis collastinensis* were ordered along a distributional gradient according to geographical location. Contrary, shapes of *Trichodactylus borellianus* were related to latitude-longitude on falling water. During these phases, the crabs of closest sites were the most similar in shape. Similar observations have been reported for *Macrobrachium vollenhovenii* (Herklots, 1851), for which morphological variations between populations were a function of distance between four rivers in Côte d’Ivoire (Konan et al. 2010). Therefore, morphometric analysis proved to be an important tool for evaluating patterns of shape variation for invertebrates by geographical location (Alibert et al. 2001, Krapivka et al. 2007).

Thus, this relationship between shape and latitudinal-longitudinal (distributional) gradient could be affected by the hydrological connectivity between rivers and by the dynamics of the floodplain system. Studies of morphological variation can elucidate patterns observed in phenotypic and genetic characteristics among populations (O’Reilly and Horn 2004). For example, the low morphological and geographical differentiation for the decapod crab *Pachygrapsus marmoratus* (Fabricius 1787) was attributable to open gene flow and consequent homogenisation (Silva et al. 2009). Additionally, a study of the crab *Carcinus maenas* (Linnaeus 1758) suggested that there was a high degree of connectivity with little evidence of reduced gene flow (Silva et al. 2010).

In addition, we found some covariation between shape and environmental variables for *Zilchiopsis collastinensis* during periods of falling water levels. However, the general pattern observed in this study showed that shape was not related to environmental variables for both species of crabs. In floodplain systems, environmental variables are affected by hydrological fluctuations together with hydrological connectivity. These events can regulate population dynamics and constitute an important macrofactor that regulates other environmental variables and can explain the distribution and abundance of organisms that live in these systems (Ward 1989, Neiff et al. 2001). On the other hand, gene flow among populations can counteract gene frequency changes because of selection, imposing a limit on local adaptation. Migration generally has an important role in evolution, affecting spatial patterns and adaptation to local environments (Hellberg et al. 2002).

In this study, we found that the two species demonstrated particular shape variations in relation to geographical location for the two periods in the hydrologic regime. This pattern can be explained by the different behaviours and life histories of each crab. For instance, *Zilchiopsis collastinensis* is a large burrowing crab that spends most of its life on the banks of rivers in canyons (Williner et al. 2009). *Trichodactylus borellianus* is a small crab that inhabits water hyacinth roots, and this species’ movements depend on macrophyte migrations (Collins et al. 2006). This indicates that these crabs could have different dispersal rates, as previously suggested by Bohonak and Jenkins (2003) for invertebrates.

Despite the shape differences found for both crab species during the two periods in the hydrologic regime, shape was more similar for individuals in downstream locality where rivers converge during periods of rising water levels. This suggests that there were exchanges in organisms along the upstream-downstream gradient, referred to as a longitudinal connection (Ward 1989).

These results showed that each freshwater crab species (*Zilchiopsis collastinensis* and *Trichodactylus borellianus*) from different localities of the middle Paraná River were connected; however, the flow of organisms changed at different phases of the hydrologic regime. More precisely, this is indicative of a specific type of hydrological connectivity (in an ecological context) that results in the water-mediated transfer of matter, energy and organisms within or between elements of the hydrologic cycle. These connections between crabs of localities can change as a function of the hydrologic regime in a floodplain system. This alteration in connectivity is to be expected because hydrological connectivity operates in longitudinal, lateral, vertical and temporal dimensions (Ward 1989), although the vertical and lateral connections were not evaluated in this study. Furthermore, the distinctiveness of floodplain macrosystems is that the level of water affects the dynamics and the relationships among populations (Poff et al. 1997, Neiff et al. 2001).

Even though this study explored the use of geometric morphometrics at a microgeographical scale, genetic analyses are required to better understand the processes of dispersal and population connectivity of freshwater crabs in this dynamic floodplain system. However, the findings of this study are particularly relevant in the context of ecological flows. When rivers are altered by a human activity, a floodplain’s hydrologic dynamics might help to maintain the ecological integrity of decapods, influencing the flow and population connectivity.
